# Application of Photoactive Compounds in Cancer Theranostics: Review on Recent Trends from Photoactive Chemistry to Artificial Intelligence

**DOI:** 10.3390/molecules29133164

**Published:** 2024-07-03

**Authors:** Patryk Szymaszek, Małgorzata Tyszka-Czochara, Joanna Ortyl

**Affiliations:** 1 Department of Biotechnology and Physical Chemistry, Faculty of Chemical Engineering and Technology, Cracow University of Technology, Warszawska 24, 31-155 Kraków, Poland; patryk.szymaszek@doktorant.pk.edu.pl; 2 Faculty of Pharmacy, Jagiellonian University Medical College, 30-688 Kraków, Poland; 3 Photo HiTech Ltd., Bobrzyńskiego 14, 30-348 Kraków, Poland; 4 Photo4Chem Ltd., Juliusza Lea 114/416A-B, 31-133 Cracow, Poland

**Keywords:** photoactive agents, cancer, theranostics, artificial intelligence, PDT, PTT, AI

## Abstract

According to the World Health Organization (WHO) and the International Agency for Research on Cancer (IARC), the number of cancer cases and deaths worldwide is predicted to nearly double by 2030, reaching 21.7 million cases and 13 million fatalities. The increase in cancer mortality is due to limitations in the diagnosis and treatment options that are currently available. The close relationship between diagnostics and medicine has made it possible for cancer patients to receive precise diagnoses and individualized care. This article discusses newly developed compounds with potential for photodynamic therapy and diagnostic applications, as well as those already in use. In addition, it discusses the use of artificial intelligence in the analysis of diagnostic images obtained using, among other things, theranostic agents.

## 1. Introduction

The current state of detection and treatment choices is limited, which is contributing to the rise in cancer mortality. Lack of public information and the onset of mild, non-specific symptoms postpone the early detection of cancer [1]. Furthermore, to confirm the diagnosis of cancer, tissue biopsies and/or fine needle aspiration cytology (FNAC) of cells from a suspected tumour are required. There is a chance that malignant cells from FNAC and biopsies will break out from the tumour and travel through the blood or interstitial fluid to other body areas [2]. Many cancer treatments, such as surgery, chemotherapy, radiation therapy, and immunotherapy, as well as targeted therapies like growth signal inhibitors, endogenous angiogenesis inhibitors, and apoptosis-inducing drugs, still have problems with toxicity against non-malignant cells and the recurrence of secondary cancers [3].

A new medical term known as cancer theranostics was created by fusing the concepts of therapies and diagnostics [4]. Because of the close relationship between medicines and diagnostics, cancer patients can now receive tailored care and accurate diagnoses. Theranostics must be able to provide the following benefits: The ability to (a) swiftly implement targeted, tailored care; (b) track and evaluate the effectiveness of anticancer drugs against tumours in patients after therapy; and (c) simultaneously diagnose and treat patients with early-stage cancer [4]. Consequently, there may be a chance of lowering the death toll from cancer, especially in cases of advanced carcinoma.

Three features of theranostic agents are often observed: imaging, tumour targeting, and therapy [5]. The ability to image and kill tumour cells makes photosensitizers with fluorescence emission and phototoxicity valuable theranostic drugs for imaging and therapy. The therapeutic role of a photosensitizer can be achieved by photothermal therapy (PTT) or photodynamic therapy (PDT) [6]. PDT uses reactive oxygen species (ROS) to damage cancer cells, whereas PTT uses heat instead of light to kill cancer cells. Some photosensitizers have built-in tumour-targeting properties because of their structural makeup. Photosensitizers with cancer-targeted units make the three benefits of theranostic drugs possible: tumour targeting, imaging, and therapy [7].

**Figure 1:**
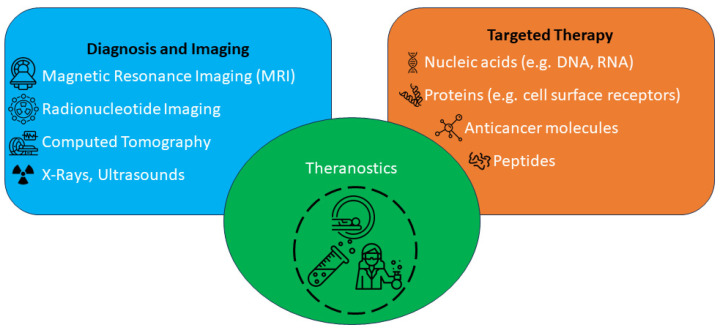
The simultaneous use of medicines and diagnostics for a cancer patient is known as cancer theranostics.

Computed tomography (CT) scans, magnetic resonance imaging (MRI), radionucleotide imaging, ultrasound, and X-rays are examples of modern imaging technologies that substantially impact cancer diagnosis and therapy in clinical settings. However, these imaging techniques are not able to identify and detect cancer cell surface markers and malignant cells for targeted cancer treatment [8]. Thus, creating new imaging techniques that use extremely sensitive probes is crucial for the accurate diagnosis and treatment of cancer. Even though they are still in the early stages of development, therapeutic agents like quantum dots, radioisotopes, and liposomes can potentially improve cancer patients’ diagnosis, treatment, and management. These agents can be bound to anticancer medications, cancer cell markers, and imaging agents with the help of available imaging techniques (Figure 1) [8].

This review discusses the usage and application of potential cancer theranostic agents. Based on several in vitro and in vivo investigations, this review addresses the benefits of targeting agents and anticancer drugs as theranostic agents. It also covers the application of artificial intelligence (AI) for diagnosis and treatment.

## 2. Design of Organic Small Molecule Photosensitizer-Based Theranostic Drugs

**Figure 2:**
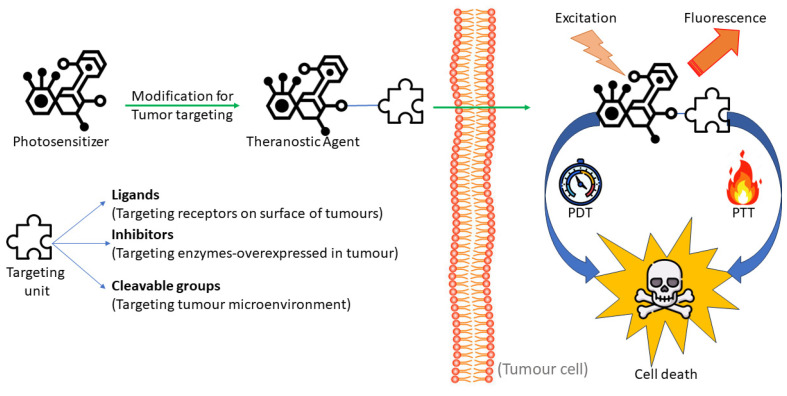
Schematic diagram of design strategies used to develop photosensitizer-based molecular theranostic agents.

Precise tumour targeting is a prerequisite for theranostics, which combines diagnostic and treatment (Figure 2). Therefore, after photosensitisers are delivered precisely, theranostics of the tumour ought to be feasible. Photosensitisers are agents that absorb specific light wavelengths and transform them into energy that can be used. In PDT, or photoactivatable therapy, cytotoxic chemicals are produced when photosensitisers, light, and molecular oxygen combine. As a result, cells died. Nonetheless, combining photosensitisers and tumour-targeting units is customary when creating theranostic drugs. Targeting units could be molecules like biotin, folic acid (FA), galactose, and the RGD (Arg-Gly-Asp) peptide sequence that attach to receptors on the surface of tumour cells. The Arg-Gly-Asp (RDG) peptide sequence supports cell attachment, and thus plays a crucial role in cell survival. Tumour-targeting units can be created by modifying photosensitisers and cleavable groups that react with the tumour microenvironment. The microenvironment of tumour cells, which differs from that of normal cells, is typically considered when creating these cleavable groupings. Therapeutic agents typically quench imaging signals and/or cytotoxicity before being activated at the tumour site. As a result, theranostic agents bearing cleavable groups are also known as activatable theranostic prodrugs. Furthermore, small molecules that target overexpressed enzymes in tumour cells may also be considered convenient targets (“targeting units”) for developing theranostic drugs. Ideally, photosensitisers might be used as theranostic agents without additional tinkering because many of them naturally target tumours [7].

## 3. Design of Theranostic Agents Using Photosensitisers and Tumour-Targeted Units

Highly specialised receptors on the surface of tumour cells, expressed intracellular enzymes, and, most notably, an increased tolerance for intracellular oxidative stress are the unique features that set tumour cells apart from normal cells [9]. In the study of tumours, some examples of receptors that have been used as tumour-targeting groups to functionalise tiny chemicals or nanomaterials are the αvβ3 integrin cellular receptors for extracellular matrix proteins, the biotin receptor, and the folate receptor (FR). Compared to their normal counterparts, these receptors are expressed more often on the cell surfaces of tumour cells [10,11,12,13,14,15]. Drugs that specifically target tumours have been created using such differences between tumour and normal cells [16]. For instance, the cysteine protease Cathepsin B, which is mostly present in the lysosomes of normal cells, may be a promising cellular target since it is expressed on the plasma membrane of many transformed cells and its level is correlated with the tumour cells’ propensity to spread. When integrin signalling is blocked, tumour development, angiogenesis, and metastasis are all inhibited [16].

## 4. Application of Photosensitisers in Theranostic Agents

### 4.1. BODIPY-Based Small Molecule Theranostic Agents

**Figure 3:**
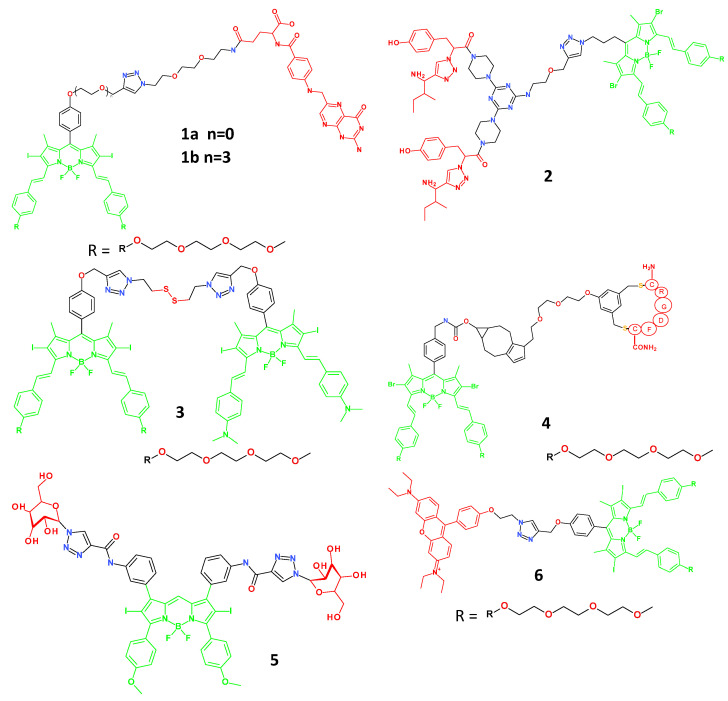
BODIPY-based small molecule theranostic agents **1**–**6**. Target units are displayed in red, and therapeutic and imaging units are in green.

Boron dipyrromethene (BODIPY) derivatives are interesting photosensitisers for developing anticancer theranostic medicines because of their desirable photophysical properties and relatively high stability, even in water settings. Ke et al. conjugated FA with different lengths of oligoethylene glycol as a linker to create two theranostic agents (Figure 3) [17]. In comparison to MCF-7 cells with low FR expression, agents **1a** (λ_max-em_ = 687 nm) and **1b** (λ_max-em_ = 689 nm) showed greater fluorescence intensity and photocytotoxicity toward epidermal carcinoma KB cells expressing the folate receptor. Interestingly, agent **1a**, which had a shorter linker than agent **1b**, demonstrated better selectivity towards KB cells (43-fold vs. 6-fold). Additionally, compound **1b** showed a strong affinity for lysosomes, whereas agent **1a** largely resided in the endoplasmic reticulum of the KB cells. This study suggests that minute structural variations in theranostic substances may influence the effectiveness of the diagnostic and therapeutic procedures [17,18,19,20].

Theranostic agent **2** (λ_max-em_ = 540 nm) was studied in experiments performed by Kamkaew et al. This molecule contains a fragment that targets the tropomyosin receptor kinase C (TrkC) receptor and a BODIPY photosensitiser that serves as a PDT agent [21]. Overexpression of TrkC receptors has been observed in some cancers, such as neuroblastoma, medulloblastoma, and breast cancer [22]. Agent **2** preferentially concentrates in TrkC-positive cells exhibiting increased photocytotoxicity, as demonstrated by in vitro tests using NIH3T3-TrkC and SY5Y neuroblastoma lines as well as the NIH3T3 Met-WT non-cancerous cell line.

Differences in the macromolecule content inside these cells can be used to improve the selective penetration of BODIPY compounds into cancer cells. Cao et al. used two BODIPY derivatives to create GSH-responsive theranostic agent **3** (λ_max-em_ = 742 nm) as the photosensitising fluorophore and quencher [23]. Since tumours elevate the ROS threshold, cellular defence against oxidative stress also increases. Among other factors, it increases the concentration of intercellular glutathione. In tumour cells (A549, HeLa, and H22) with a relatively high concentration of GSH, agent **3** fluoresced brightly. In contrast, significantly less fluorescence was observed in HELF cells with less GSH. Substantial tumour growth suppression and effective imaging capabilities of agent **3** were demonstrated in an in vivo study using mice with transplanted H22 hepatoma tumours. The cyclic RGD (cRGD) moiety serves as the tumour-targeting component of theranostic agent **4** (λ_max-em_ = 696 nm) [24,25]. The v3-positive cancer cell lines U87-MG and A549 vs. MCF-7 displayed significant and selective affinities for agent **4**, and agent **4** also successfully reduced the growth of v3-overexpressed tumours in vivo. To achieve precise targeting, theranostic molecule **5** (λ_max-em_ = 726 nm) was designed and targeted against the high expression of the glucose transporter GLUT1, which occurs on the surface of highly proliferating tumour cells depending on glucose as a preferential energy source [26]. Compared to normal cells (HFL1), agent **5** showed greater fluorescence intensity, interacting with MDA-MB-231 and MCF-7 tumour cells. Moreover, only a brief (1 min) irradiation was necessary to destroy tumour cells selectively. Substantial phototoxicity was observed in normal cells with longer (5 min) irradiation. With a cationic rhodamine moiety serving as the energy donor and the photosensitiser diiododistyrylbodipy (BDP) serving as the energy acceptor, small-molecule **6** (λ_max-em_ = 687 nm) was a highly efficient theranostic agent based on the Förster Resonance Energy Transfer (FRET) mechanism [27]. The existence of a positive charge prompts an innate tumour-targeting property that allows it to accumulate more frequently in tumours. It lights up after intravenous injection, separates tumour sites from surrounding tissues, and displays a signal-to-background ratio of up to 12.5. An in vivo study showed that using molecule **6** entirely stopped the development of the tumour and increased the lifespan of mice.

### 4.2. Porphyrin-Based Small Molecule Theranostic Agents

**Figure 4:**
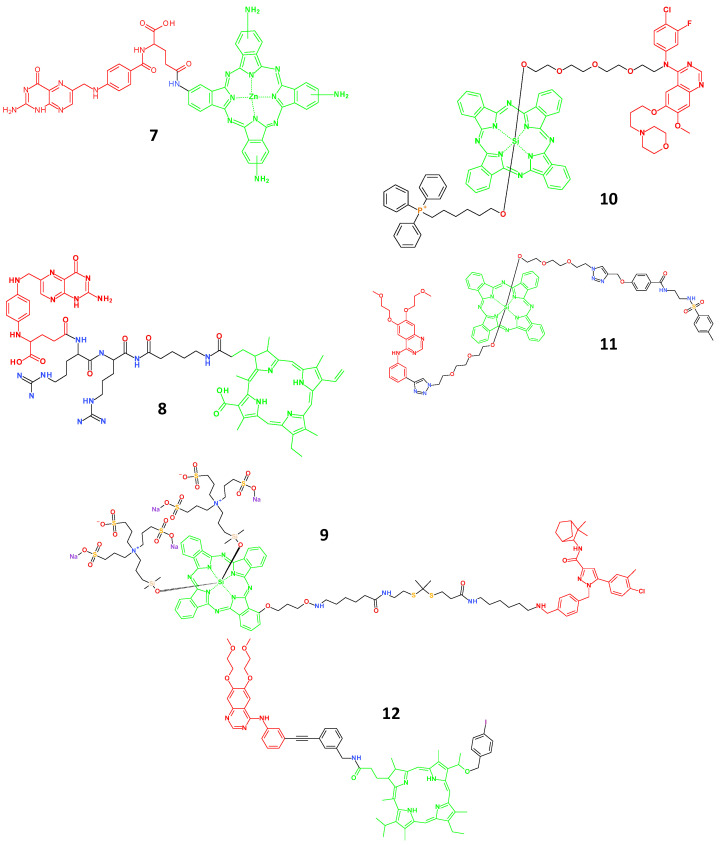
Porphyrin-based small molecule theranostic agents **7**–**12**. The target, therapeutic, and imaging units are displayed in red, blue, and green, respectively.

Because their skeletons are comparable to those of phthalocyanine derivatives, porphyrins are regarded as the first generation of photosensitisers. However, they are currently extensively used in the design of theranostic agents (Figure 4). The theranostic agent **7** (λ_max-em_ = 740 nm), which Wang et al. created by conjugating FA to zinc tetraaminophthalocyanine, demonstrated a particular affinity for FR-overexpressing KB cells, as opposed to FR-negative A549 cells [28]. Additionally, under two-photon excitation of a near-infrared (NIR) laser, agent **7** demonstrated PDT efficacy that was ten times greater than that of sulfonated aluminium phthalocyanine, an approved photosensitiser for clinical use. By using a di-arginine linker to combine the photosensitiser chlorin e4 (Ce4) and tumour-targeting unit FA, Kim et al. improved this design for theranostic agents **8** (λ_max-em_ = 670 nm) [29,30]. Lysosomal cathepsin B binds to di-arginine residues and is overexpressed in some tumour cells [31]. As a result, theranostic agent **8** only produced NIR fluorescence imaging and PDT therapy in cancer cells that were FR-positive and overexpressing cathepsin B. Additionally, this dual-targeted approach boosted the tumour suppression impact, decreased unwanted phototoxic effects, and raised the target-to-background ratio in in vivo imaging.

Ling et al. created theranostic agent **9** (λ_max-em_ = 690 nm) in a different study, which consisted of a phthalocyanine photosensitiser, ROS-sensitive linker, and cannabinoid anticancer medication [32]. Targeting the type-2 cannabinoid receptor, which is overexpressed in cancer cells, allows it to indicate tumour cells specifically. Agent **9** also showed significant therapeutic efficacy in type-2 cannabinoid receptor-expressing DBT-2D4 cells, gained from the combined use of cannabinoids and PDT therapy. Using silicon phthalocyanine as the photosensitiser and EGFR inhibitors as the tumour-targeting component, Xue et al. created theranostic agents **10** (λ_max-em_ = 680 nm) and **11** (λ_max-em_ = 674 nm) [33,34]. They identified and elucidated the death of tumour cells overexpressing EGFR. Agents **10** and **11** were localised in different cellular compartments; agent **10** was found in the mitochondria, whereas agent **11** was localised in the endoplasmic reticulum. Agent **11** demonstrated superior imaging capabilities for A549 cells with high EGFR expression compared with HELF cells with low EGFR expression. Both substances also showed exceptional photodynamic tumour ablation capabilities with few negative effects. By combining the photosensitiser porphyrin, the EGFR inhibitor erlotinib, and the positron emission tomography (PET) agent (^127^I), Cheruku et al. designed and tested theranostic agent **12** (λ_max-em_ = 673 nm) [35,36] After imaging it using PET, it provided the option to use fluorescence-guided PDT to treat bladder cancers.

### 4.3. Small Molecule Theranostic Agents Based on Aggregation-Induced Emission (AIE)

**Figure 5:**
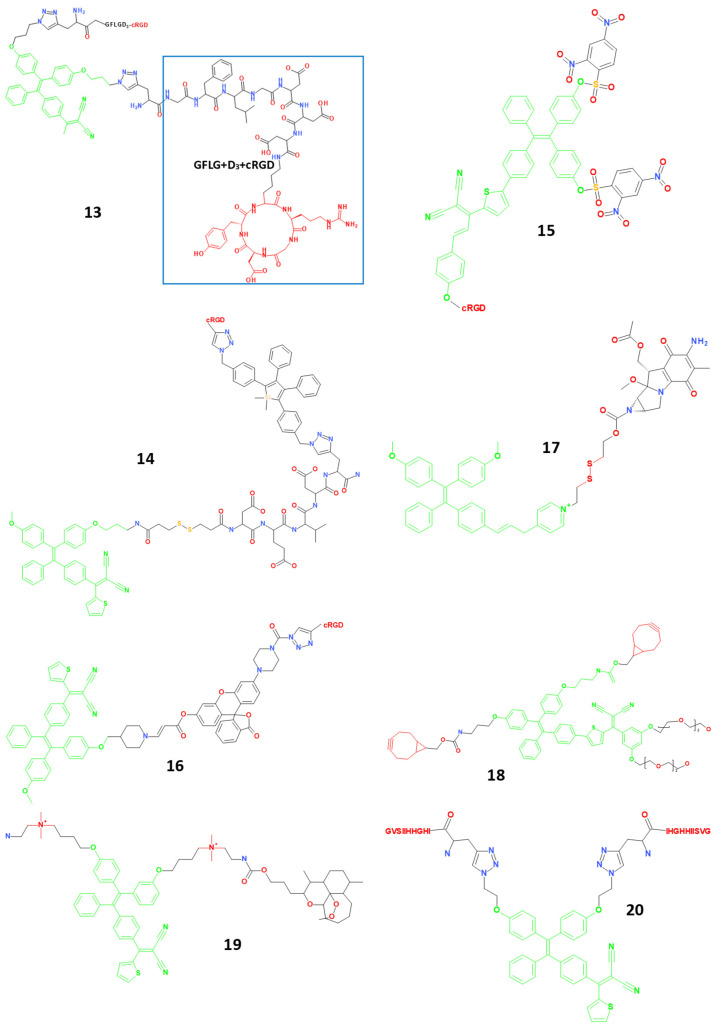

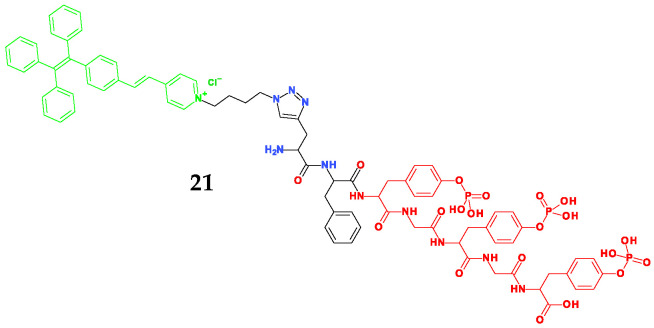
AIE-based small molecule theranostic agents **13**–**21**. The target, therapeutic, and imaging units are displayed in red, blue, and green, respectively.

Due to their intense fluorescence and quick ROS formation in the aggregated state, photosensitizers with aggregation-induced emission (AIE) characteristics have been investigated to develop theranostic agents (Figure 5). This approach allows increased fluorescence intensity and sensitivity. Several theranostic agents **13**–**19** (λ_max-em_ = 13: 613 nm, 14: 650 nm, 15: 650 nm, 16: 645 nm, 17: 660 nm, 18: 685 nm, 19: 365 nm) based on AIE photosensitizers were reported by Liu et al. [37,38,39,40,41,42,43]. AIE fluorogen with dicyanovinyl is an imaging reagent and photosensitizer, a Gly-Phe-Leu-Gly (GFLG) peptide responding to cathepsin B, and a hydrophilic linker with three Asp (D) residues to increase the hydrophilicity of the four components of the theranostic agent 13 [37,44]. Theoretically, the GFLG sequence in agent **13** might be cleaved by cathepsin B overexpressed in tumours after being transported to cancer cells with overexpressed αvβ3 integrin receptors, resulting in an enhanced fluorescence signal output concurrent with activated photoactivity for image-guided PDT. Compared to MCF-7 and 293T cells, which served as the negative controls, agent **13** increased red fluorescence and had a greater PDT efficiency in MDA-MB-231 cells overexpressing the αvβ3 integrin receptor (Figure 5). The RGD targeting group and red-emissive AIE photosensitizer were combined to create theranostic agent **14** [38]. Adding an apoptosis sensor that can be activated by caspase-3/-7 to produce bright green fluorescence gave agent **14** a new function of real-time therapeutic response monitoring, better than agent **13**. As a result, **14** is particularly practical for theranostics because it can simultaneously gather the green emission from the apoptosis sensor and the red fluorescence from the photosensitizer with a single wavelength stimulation. Theranostic agent **15** used a tetraphenylethylene derivative as a photosensitizer and also used as a tumour-targeting unit. In addition, it was embellished with a quencher moiety called 2,4-dinitrobenzene sulfonyl, which can quench the photosensitizer’s fluorescence and photosensitizing activity and can be cleaved by high levels of GSH in tumour cells [39,45]. Agent **15** was able to eliminate tumour cells, which enhanced the target-to-background ratio for cancer therapy. The theranostic agent **16** used the same photosensitizer as agent **14** and added a fluorogenic green emissive rhodol dye via a singlet oxygen-cleavable aminoacrylate linker. This allowed agent **16** to monitor singlet oxygen generation in situ and in real-time, which advanced the method for assessing the therapeutic effect early on. A DNA cross-linking anticancer drug, Mitomycin C, together with vinyl pyridinium-substituted tetraphenylethylene was linked via a tumour-responsive disulfide bond in agent **17**, a dual-prodrug GSH-activatable theranostic agent [40,46]. Mitomycin C suppressed the photosensitizing and fluorescent properties of agent **17**, and an electron-withdrawing acyl at the nitrogen position close to the linker reduced the cytotoxicity of Mitomycin C. Agent **17** can simultaneously release the active photosensitiser and Mitomycin C for combined therapy and tumour-specific imaging upon GSH activation. A study using four T1 tumour-bearing animals in vivo revealed that agent **17** significantly inhibited tumour growth and expressed the capacity to image the tumour site precisely. Theranostic agent **18** delivered a photosensitizer to the tumour site using a bioorthogonal tumour labelling method for tumour-specific imaging and PDT [42,47,48]. First, using metabolic engineering, the cell membrane glycoprotein was changed with azide groups using acetylsialic acid, which had undergone azide modification. Subsequently, **18** was added to the cells, causing a bioorthogonal reaction. Agent **18** was linked to the surface of tumour cells because of the enhanced fluorescence signal on these cells. Azide-modified acetylsialic acid was pre-injected into tumour-bearing mice, and agent **18** demonstrated excellent tumour labelling and therapeutic potential in a rodent model. Tetraphenylethenethiophene (which served as a fluorescent reporter and photosensitizer), two quaternary ammonium salts, and the anticancer medication artemisinin were used as theranostic agents **19** [43]. Due to increased cell plasma and mitochondrial membrane potentials in tumour cells compared to normal cells, the quaternary ammonium salt-loaded agent **19** accumulates predominantly in the mitochondria of tumour cells.

A synergistic effect between the PDT of tetraphenylethenethiophene and artemisinin chemotherapy increased the effectiveness of cancer cell treatment using agent **19**.

Hu et al. synthesized theranostic agent **20** (λ_max-em_ = 630 nm) with a cancer cell-specific peptide (GVSIIHHGHI) as the targeting group and an AIE fluorophore acting as a photosensitizer [49,50]. This is due to the ability of the peptide to bind to lysosomal-associated transmembrane protein 4 B (LAPTM4B), an overexpressed protein in most solid tumours. Therefore, agent **20** has high selectivity towards tumour cells. Agent **20**’s phototoxicity to cancer cells (HepG2, HeLa, and U2OS) and non-toxicity to normal cells (HEK293 cells) proved its specific photodynamic therapy. Theranostic agent **21** (λ_max-em_ = 600 nm), created by Ji et al., contains an AIE fluorophore and a short peptide with three tyrosine phosphates. It has excellent fluorescence visualisation and PDT capability for alkaline phosphatase-overexpressing cancer cells (Saos-2 cells vs. HEK-293 T cells) compared to normal cells [51,52]. These experiments demonstrated the high efficiency of theranostic agents for selective aggregation in tumour cells [51,53].

### 4.4. Theranostic Agents Responding to ROS or H2S

**Figure 6:**
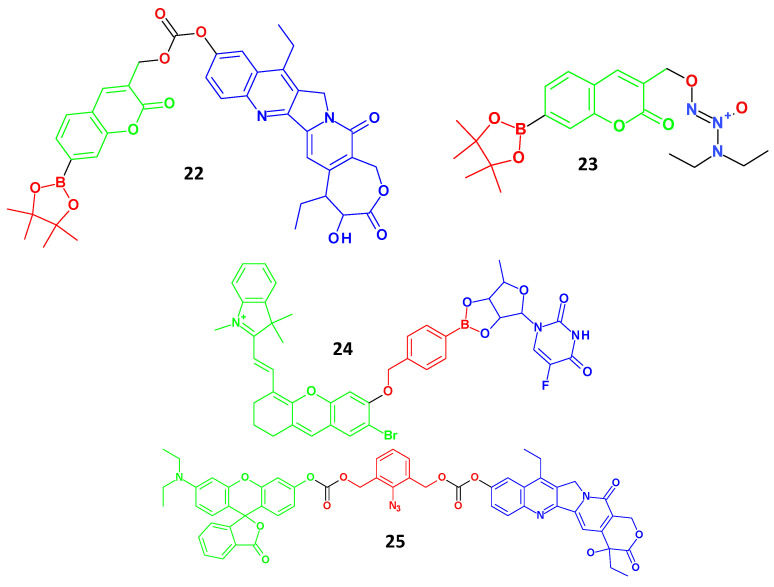
Tumour intracellular environment-driven tumour-targeting theranostic agents **22**–**25**. Target, therapeutic, and imaging units are displayed in red, blue, and green.

Elevated ROS levels characterise most tumour cells compared with those in normal cells. Increased concentrations of ROS, such as H_2_O_2,_ can be used for cancer therapy using theranostic drugs. Theranostic molecules are not drugs but prodrugs because they do not provoke cell death after the activation of ROS or H_2_S. Theranostic prodrugs can be activated by high quantities of ROS, such as H_2_O_2_, found in various tumour cells. In line with this, Kim et al. created an activatable theranostic prodrug based on high levels of H_2_O_2_ dedicated to metastatic lung malignant cells [54]. A coumarin unit was employed as the fluorophore to track tumour cells in agent **22** (λ_max-em_ = 600 nm), a boronate ester was used as the cleavable group responding to H_2_O_2_, and a camptothecin derivative, SN-38, was used as the chemotherapeutic component. In a mouse model of metastatic lung disease, agent **22** selectively reduced tumour cell proliferation and demonstrated effective anticancer efficacy (Figure 6).

In another study, Ravikumar et al. combined boronate ester, coumarin, and nitric oxide (NO) donor to create a theranostic prodrug **23** (λ_max-em_ = 460 nm) [55]. The increased fluorescence signal in tumour cells demonstrated that agent **23** was specifically activated in tumour cells with high ROS levels. After activation, agent **23** released NO, causing DNA damage and resulting in cell death. Moreover, agent **23** had a notable cytotoxic effect on tumour cells but a negligible cytotoxic effect on normal cells. By employing a boronate ester group as the linker to connect the NIR fluorescence monitor to the anticancer medication 5′-DFUR, Liu et al. created a theranostic prodrug **24** (λ_max-em_ = 710 nm) [56]. The boronate ester group was broken to produce 5′-DFUR for chemotherapy and an active NIR fluorescent dye for tumour cell monitoring, taking advantage of the high H_2_O_2_ levels in tumour cells. The NIR fluorescent dye utilised in this study is also a photosensitiser, which can have a synergistic chemo-photodynamic therapeutic effect to increase the cytotoxicity of cancer cells. In nude mice with tumours, agent **24** demonstrated excellent tumour-targeting and inhibitory actions (Figure 6).

Given that cystathionine-lyase and cystathionine-synthase, responsible for generating H_2_S, are overexpressed in some cancer cells, endogenous H_2_S is considered a special stimulator for delivering chemotherapeutics. The theranostic prodrug **25** (λ_max-em_ = 549 nm), which was conjugated with rhodamine as the imaging group and SN-38 as the therapeutic agent, was created by Bobba et al. using an azide benzyl-carbonate moiety as the linker in response to H_2_S [57]. Agent **25** showed higher fluorescence intensity and increased antiproliferative activity in tumour cells (HCT116 and A549 cells) than in normal cells (WI-38 cells), which enabled imaging diagnosis and targeted tumour therapy. Agent **25** was preferentially activated in tumour cells, leading to the release of anti-tumour drugs and fluorophores.

### 4.5. Application of Complexes (III) in PDT

Ir(III) complexes exhibit exceptional brightness and photochemical stability, making them excellent candidates for bioimaging applications. They can be used to image cellular processes and the microenvironment inside single cells with unprecedented clarity, promoting an understanding of disease mechanisms at the molecular level. Ir(III) complexes can be engineered to target cancer cells selectively. Once on the target, these complexes can act as photosensitisers in PDT, generating ROS upon light activation, leading to cell death. PDT uses ROS to damage cancer cells, whereas PTT uses light-generated heat. Ir(III) complexes can act through various mechanisms such as ROS generation, interference with mitochondrial function, arrest of the cell cycle, and induction of apoptosis and autophagy. Ir(III) complexes represent a promising group of compounds for cancer therapy and diagnosis. Their ability to simultaneously image and destroy tumour cells offers the potential for more precise and personalised treatment strategies, which could help reduce mortality rates in the advanced stages of cancer.

### 4.6. Quantum Dots as Theranostic Agents

Quantum dots (QDs) are semiconducting nanocrystals formed from elements in periodic chart groups II, III, IV, V, and VI. Semiconducting nanocrystals are excellent candidates for bioimaging agents because of their capacity to glow upon excitation [4,8]. In addition, QDs are useful drug carriers for targeted cancer therapies. As a result, QDs conjugated with anticancer medications and targeted agents, along with necessary imaging techniques such as CT and MRI, could open the way for the simultaneous diagnosis and treatment of cancer simultaneously [4,58,59,60].

**Figure 7:**
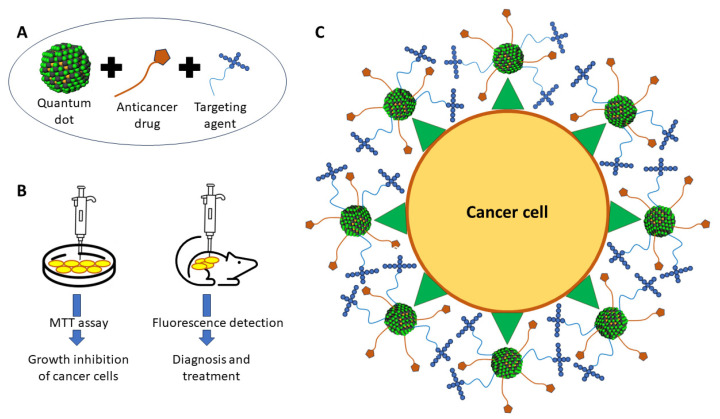
Application of quantum dots (QDs) as a theranostic agent in cancer. (**A**) QDs were linked with polypeptides (targeting agents) and anticancer drugs. (**B**) The mixture containing quantum dots, targeting agents, and anticancer drugs was introduced directly onto (i) HT-29 human colon adenocarcinoma cells and SKOV3 ovarian cancer cells. Growth inhibition of cancer cells in both experiments was observed using an MTT assay. Introduction of a mixture containing quantum dots, targeting agents, and anticancer drugs in (ii) SKOV3 ovarian tumour-bearing mice resulted in simultaneous identification and destruction of tumour cells using fluorescence imaging. (**C**) The polypeptides from the mixture bind to the glycosaminoglycans, which are abundantly present in cancer cells. Upon excitation, QDs fluoresce and act as an imaging agent for cancer diagnosis. The anticancer drug in the mixture encourages targeted therapy. Overall, the ability of this mixture to be a therapeutic agent and imaging agent suggests that applying QDs with targeting agents and anticancer drugs could be a good theranostic agent for the diagnosis and treatment of cancer [66].

Brunetti et al. (2020) [60] provided a classic illustration of cancer theranostics employing QDs coupled with tetra-branched peptides as a targeting agent and anticancer medication [61]. Malignant cancer cells overexpress glycosaminoglycans, which are found on the surface of the epithelium, endothelium, and extracellular matrix. The quantity of glycosaminoglycans aids the survival of cancer cells by promoting their growth, invasion, metastasis, and angiogenesis [60,62,63]. The ability of tetra-branched polypeptides (NT4) to bind with sulphated glycosaminoglycans and endocytic receptor tumour markers, such as low-density lipoprotein receptor-related protein 1 (LRP1) and low-density lipoprotein receptor-related protein 6 (LRP6), makes it a potential target molecule for cancer cells [63]. As a result, QDs were combined with paclitaxel and the NT4 peptide (NT4-QDPTX) for targeted distribution and deliberate killing of cancer cells. Interestingly, NT4-QD-PTX demonstrated potent cytotoxic activity against HT-29 human colon cancer cells [64]. In another study, a hydrogel containing the designed coiled-coil polypeptides, Ag_2_S QDs, and paclitaxel (PC10A/Ag_2_S QD/PTX) was delivered to tumours, and anticancer efficacy was observed [65]. The PC10A/Ag_2_S QD/PTX hydrogel severely inhibited the development of SKOV3 ovarian cancer cells in vitro and in vivo [65]. The fact that PC10A/Ag_2_S QD/PTX is both a therapeutic and an imaging agent raises the possibility that this compound could be an effective theranostic agent for detecting and managing cancer (Figure 7).

Moreover, a recent study found that QDs improve the efficacy of chemo-photothermal therapy against cancer cells. When QDs and a photothermal agent, namely graphene, are combined, the heat released by near-infrared radiation causes the elimination of cancer cells. MDA-MB-231 breast cancer cells’ survival was reduced by graphene oxide-graphene quantum dots hybrids (GO-PEI-GQDs) under laser excitation in comparison to untreated cells [67]. The anticancer effect of GO-PEI-GQDs after radiation treatment emphasised the function of graphene oxide in transferring heat to eliminate cancer cells. More thorough in vivo toxicity studies are required before graphene QDs may be used in the field of cancer theranostics, even though it has been demonstrated that they are harmless and do not damage normal cells in in vitro studies. Table 1 summarises other uses of QDs for the detection and treatment of cancer [68].

### 4.7. Radioisotopes

Although radioisotopes are not light-activated, anticancer therapy and diagnostic techniques based on them are an important part of the medical anticancer arsenal. For this reason, and the fact that radioisotopes are associated with the emission of radiation, we have decided to devote a section to them in this article. The term “photoactive” generally refers to compounds that respond to light, such as absorbing photons and undergoing a chemical change or emitting light (fluorescence or phosphorescence). However, radioisotopes are characterised by their radioactive properties, meaning they emit radiation (alpha particles, beta particles, or gamma rays) due to nuclear decay processes rather than interactions with light.

**Figure 8:**
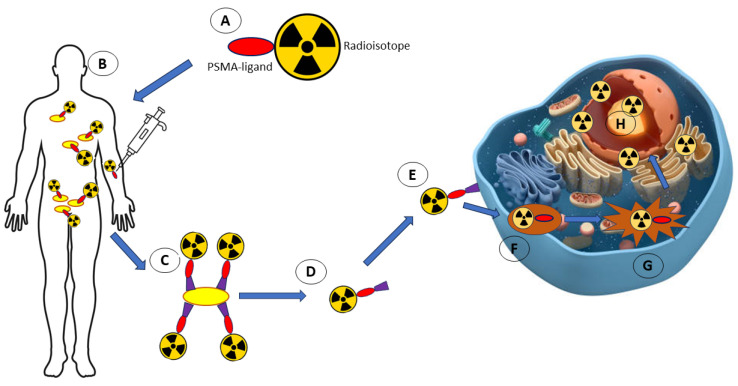
The anticancer mechanism of radioisotope combined with a ligand targeting PSMA in lethal metastatic castration-resistant prostate cancer (mCRPC) patients. (**A**,**B**) Once PSMA-ligand has been conjugated to a respective radioisotope, the radioligand is then injected into the patient. (**C**–**E**) Radioligand selectively binds to PSMA receptors on prostate cancer cells and enters the cell via endocytosis. (**F**,**G**) The radioligands continue to damage endosomes, releasing the radioligands into the surrounding matrix of the cell. (**H**) The radioligands then destroy the cancer cells by inducing damage in crucial organelles such as the nucleus and mitochondria without affecting the normal, unaffected cells. The ability of these radioligands to target specific cancer cells encourages targeted therapy and effective diagnosis via imaging techniques such as PET/CT scans [66].

For decades, it has been suggested that radioisotopes like Terbium (Tb) and lutetium (Lu) may be used as theranostic agents in the medical industry [71,72]. A valuable diagnostic tool and therapeutic alternative for standard prostate cancer therapy has recently been proposed as a combination of a radioisotope and an antagonist that targets prostate-specific membrane antigen (PSMA) [71]. This radioligand therapy uses lutetium 177 (^177^Lu)-PSMA (LuPSMA) to invade the cancer cell membrane and create a vacuole, which affects cancer cells (Figure 8). Therefore, the cytotoxic activity of radioisotopes is restricted to exposed cancer cells and does not affect adjacent normal cells. Interestingly, metastatic castration-resistant prostate cancer (mCRPC) patients treated with LuPSMA demonstrated a >50% reduction in PSA expression with few side effects [72]. A study by Müller et al. showed that a 59-year-old man with metastatic castration-resistant prostate cancer (mCRPC) who received ^152^Tb-PSMA-617 injections in a different study showed no negative side effects or toxicity, indicating the usefulness of ^152^Tb-PSMA-617 as a PET/CT imaging tool for the detection and identification of patients with mCRPC in clinical settings [72]. Notably, the combination of the therapeutic drug LuPSMA with imaging agent ^152^Tb-PSMA617 may be an effective theranostic agent for treating cancer patients.

Cancer cell growth and metastasis depend on angiogenesis. The binding site for angiotensin, an anti-angiogenic factor, is found on tumour cells’ surface and inner mitochondrial membranes. The effectiveness of a radioiodine-labelled anti-adenosine triphosphate synthase monoclonal antibody (I-ATPS mAb) as a possible cancer theranostic drug has been established in studies using this information. By specifically targeting ATP synthase, I-ATPS mAb reduced the development of MKN-45 gastric cancer cells and inhibited angiogenesis [73]. The capacity of cetuximab labelled with Zirconium-89 (^89^Zr) as a potential theranostic agent candidate for the treatment of locally advanced head and neck squamous cell carcinomas (LAHNSCC) was also demonstrated in another study. PET/CT imaging was used to confirm the uptake of cetuximab labelled with ^89^Zr in LAHNSCC tumour cells and lymph nodes [74]. Cetuximab labelled with ^89^Zr can target cancer cells and be an effective imaging agent, and thorough research could result in the creation of novel potential cancer theranostic medicines.

### 4.8. Liposomes

**Figure 9:**
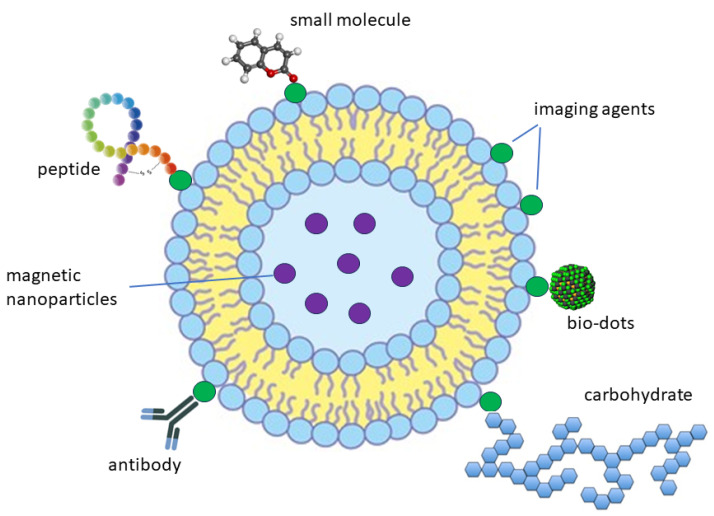
Liposomes tolerate conjugation with numerous targeting ligands for targeted therapy. Numerous targeting agents such as carbohydrates, peptides, small molecules, biodots (e.g., DNA biodots, quantum biodots) and antibodies can be conjugated to a liposome. These conjugated liposomes could be viewed by attaching imaging agents and/or magnetic nanoparticles. The ability of liposomes to be conjugated to various targeting agents, anticancer molecules, and imaging agents makes it a good theranostic agent [66].

Liposomes are spherical vesicles with a lipid bilayer that are well known for their capacity to encapsulate and protect encapsulated anticancer molecules and diagnostic agents, extend the half-life of transported molecules, and tolerate conjugation with a variety of targeting ligands for targeted therapy [75,76]. Magnetic resonance imaging (MRI) is frequently employed for morphological observations using contrast agents such as gadolinium (Gd). As it has been demonstrated that liposomes complexed with Gd have no toxicity or adverse effects on normal cells, these complexes, once filled with anticancer compounds and coupled with particular ligands of interest, could serve as possible theranostic agents for the treatment of specific cancers (Figure 9) [76].

The effectiveness of targeted liposomes co-loaded and conjugated with DNA biodots, etoposide, and cetuximab (CTXBD-ETP liposomes) in the treatment of advanced-stage non-small cell lung carcinoma (NSCLC) was demonstrated by Jha et al. Targeting EGFR in A549 lung cancer cells showed that the CTXBD-ETP liposomes displayed potent cytotoxic activity against these cells [77]. It has also been demonstrated that photosensitive liposomes can selectively kill cancer cells upon irradiation without damaging neighbouring cells. TPCI and paclitaxel-encapsulated liposomes resulted in a decrease in prostate cancer tumour cells. The initiation of apoptosis in PC3 cells is caused by the release of TPCI in cancer cells [78].

Accumulating evidence suggests that several Glucose Transporters (GLUT) are overexpressed in cancer cells, including in gastric cancer cells. Ginsenoside interacts with GLUT in tumour cells; hence, it may be utilised as a target ligand to help drug-loaded liposomes accumulate in the intended tumour. By causing cell cycle arrest in the G2/M phase and inducing apoptosis, ginsenoside paclitaxel-liposomes inhibited the development of BGC-823 human gastric cancer cells. The impact of ginsenoside paclitaxel liposomes on normal cells, however, was not specified [79].

## 5. Artificial Intelligence for Cancer Theranostics

Cancer is a leading cause of death worldwide. As an illustration, the number of new cases of cancer rose from 14 million in 2012 to 19.3 million in 2020, while the number of cancer fatalities grew from 8.2 million in 2012 to roughly 10 million in 2020 [80,81]. The survival rate and quality of life of cancer patients can be greatly increased by early cancer detection, correct diagnosis, and high-efficiency cancer therapy with low side effects. Super-resolution microscopy imaging, optical endoscopy imaging, optical coherence tomography, photoacoustic imaging, diffuse optical tomography, Raman spectroscopy imaging, and fluorescence imaging are optical imaging techniques that include various microscopy and tomography imaging methods at various scales. Interestingly, they have drawn much attention in preclinical and clinical cancer diagnosis and therapy research. Optical imaging has proven to have unbeatable advantages for cancer oncology when compared to conventional imaging techniques, such as positron emission tomography (PET), computed tomography (CT), and magnetic resonance imaging (MRI), including high sensitivity, low cost, and the ability to visualise both structural and functional information of tumour tissues at different scales [82,83,84]. Additionally, many optical contrasts, including absorption, scattering, and fluences, which are related to the characteristics of cancer, are the foundation of several optical imaging techniques. For instance, photoacoustic imaging is based on the optical absorption of biological tissues, whereas Raman and optical coherence tomography imaging depend on light scattering. Fluorescence imaging, on the other hand, relies on light emission following its absorption by fluorescence probes. The low penetration depth of optical imaging is a drawback that can be overcome using endoscopic methods. Additionally, it was shown that the second near-infrared (NIR) biological window (wavelengths from 1000 to 1700 nm) allows optical imaging to penetrate as deep as a few centimetres because the skin and blood scatter and absorb less light at long wavelengths [85,86,87,88].

**Figure 10:**
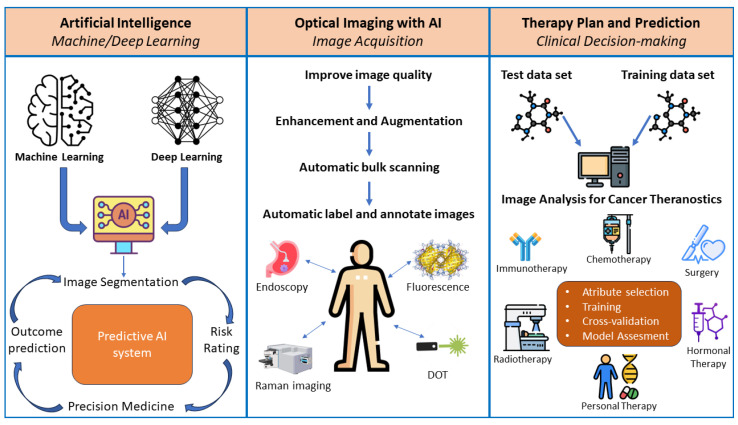
Overview of optical imaging with artificial intelligence (AI) support for accurate cancer theranostics [97].

In recent years, optical imaging has gradually transitioned from the classic imaging mode to an era of computational and intelligent optical imaging [89,90,91,92,93]. The imaging mode that uses a camera system to capture the specimen directly has increasingly been applied to the indirect imaging mode, which calculates and reconstructs images using the data gathered. Cancer diagnosis and imaging-guided therapy have greatly benefited from the application of AI in pathological diagnosis and medical image recognition. For instance, computer vision, a common AI technique, can assist in accurately identifying specific elements in pictures for diagnosing cancer. By processing and cross-referencing health and medical big data, including pictures, pathology, and genes, a significant quantity of pathological and genetic data can be mined, making it easier to access the pathological sections quickly and improve the effectiveness and prognosis of cancer diagnosis. A unique issue posed by biopsy, an intrusive, unpredictable method that typically ignores heterogeneity within cancer, has been largely resolved by recent advancements in the medical imaging of cancer. AI uses data characterisation techniques to transform regular imaging data into disease biomarkers. These artificial intelligence techniques have been applied in histopathological image interpretation, medication development, bioinformatics, genome sequencing, and radiological diagnosis. It is interesting to note that AI has demonstrated the capability of performing histological diagnoses on par with experts in medicine. Additionally, AI-assisted optical imaging may access information in multidimensional, high temporal–spatial resolution that conventional microscopy techniques cannot or find it difficult to directly reach through the calculated reconstruction mode [94,95]. Among them, compressive sensing technology, represented by physical-model-driven and data-driven deep learning (DL) technology, improved the unpredictable nature of the actual imaging physical process and the difficulty of solving high-dimensional ill-posed inverse problems [96,97,98,99,100,101], opening up new possibilities for the advancement of optical imaging (Figure 10).

**Figure 11:**
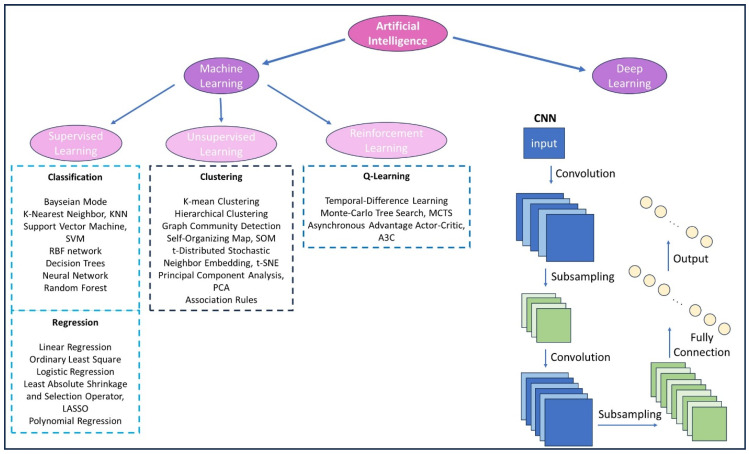
The relationship between artificial intelligence, machine learning, deep learning, and common algorithms, otherwise known as a convolutional neural network, or CNN [97].

AI is a subfield of computer science that aims to replicate intelligent human behaviour in machines. It is described as a machine configured to learn and recognise relationships and patterns between inputs and outputs and then utilise this knowledge to make decisions on fresh input data. The two main techniques for implementing AI are machine learning (ML) and deep learning (DL) (Figure 11).

When making predictions, ML algorithms rely on structured data [102], which are described as data that have been labelled, arranged, and specified with certain features. Clinical decision-making is improved at all levels using machine learning (ML) models, which analyse data and find patterns. These models update on their own and become more accurate every time [103]. ML techniques can also be divided into supervised, unsupervised, and reinforcement-learning categories. Supervised methods such as Bayesian methods, discriminant analysis, k-nearest neighbour (kNN), support vector machine (SVM), artificial neural network (ANN), random forest, AdaBoost, and fuzzy techniques use images along with ground truth labels to train classification models. Vessel segmentation is performed using unsupervised techniques such as the Gaussian mixture model (GMM), fuzzy c-means (FCM), and k-means clustering without the need for training labels. With the aid of a continuous feedback loop, reinforcement learning may be learned through a system of rewards and penalties, and its algorithm may be developed over time. DL and traditional machine learning (including rule-based learning) typically differentiate and detect cancerous images from normal tissues and healthy controls.

An approach that automatically learns tasks and features from a training dataset is called DL, a subset of machine learning. A network of interconnected algorithms is referred to as a neural network, and the term “deep” alludes to the several layers of algorithms that the provided data goes through during computing [104]. The neuronal connections in the human brain, which were built to recognise patterns in their work, inspired this. Deep learning (DL) uses deep neural networks (DNNs) to create complex models with numerous hidden layers that assess different types of data and produce predictions [105]. In addition, each DL phase enables the program to continuously learn and assess its performance to achieve a particular result. In contrast to machine learning (ML) programs, deep learning (DL) programs require numerous layers of coding and do not require a programmer to manually identify certain features in an image because DL programs automatically learn from training datasets. As a result, a DL program also requires a larger training dataset and more processing capacity than a traditional ML program. DL frameworks come in many different varieties, including autoencoders (AEs), deep belief networks (DBNs), and convolution neural networks (CNNs). The most frequently employed method for cancer detection is the CNN, followed by the AE and DBN. They either examine molecular-level data, such as gene mutations and gene expression data or evaluate medical imaging. Lung cancer, breast cancer, and other prevalent malignancies are the detection targets in the current study because DL technology cannot currently be applied to all types of cancer [106]. A CNN is a multilayer neural network framework that uses convolutional processing to learn high-level information from data. The convolutional, pooling, and fully connected layers are the three different types of neuron layers present. The pooling layer is typically used to minimise the dimension (complexity) of the data, whereas the fully connected layer utilises the knowledge gained from the first two layers for classification. The convolutional layer extracts features from the data. However, not all forms of cancer can be effectively detected and distinguished using DL technology [107,108,109].

There are numerous more types of AI technical approaches with various benefits and uses, despite the fact that multilayer perceptron (MLP), recurrent neural network (RNN), and CNN are the most fundamental and widely utilised building blocks for more advanced techniques. They are either used to evaluate molecular-level data, such as gene mutations and gene expression data, or medical images, such as X-ray and CT imaging. It has also become clear that optical imaging, particularly when combined with AI-assisted analysis methods like computer vision and natural language processing (NLP), is potent for increasing the precision and effectiveness of cancer detection, diagnosis, and treatment planning. The processing and interpretation of visual data obtained through optical imaging methods, including microscopy, endoscopy, and radiology, are made possible by computer vision, a subfield of artificial intelligence. Computer vision algorithms have transformed image analysis in the field of cancer diagnosis, facilitating the identification of questionable lesions and the segmentation and classification of tumours. Computer vision algorithms improve detection sensitivity and specificity by utilising cutting-edge image-processing techniques, helping to provide earlier and more precise cancer diagnoses. In addition, computer vision is essential for determining treatment efficacy and tracking the disease course. NLP approaches make extracting useful information from the medical literature, clinical reports, and patient data for cancer therapy easier. NLP algorithms can help with clinical trial discovery, therapeutic decision-making, and individualised patient care by evaluating large amounts of unstructured text data. To help oncologists develop effective treatment plans, NLP-powered technologies can extract pertinent information from medical texts such as research articles, clinical guidelines, and case studies. Additionally, NLP can help with automated summarisation and interpretation of patient data, lessening the cognitive burden on medical personnel and facilitating more effective and precise treatment planning. AI-assisted optical imaging has demonstrated promising outcomes in detecting and treating cancer, owing to the fusion of computer vision and NLP approaches. These methods enable a more comprehensive understanding of cancer-related materials by fusing the strength of image analysis with text mining. For instance, NLP algorithms can extract essential clinical and genetic data from patient records, whereas computer vision can extract visual elements from medical photos. These data streams can be combined to create more thorough and individualised diagnostic reports, empowering medical professionals to choose the best course of action. Automated summary and interpretation of patient data is another area in which NLP can be used. This reduces the cognitive load on the medical staff and enables more effective and precise treatment planning. The combination of computer vision and NLP techniques has yielded promising results in detecting and treating cancer using AI-assisted optical imaging. These techniques combine the advantages of text mining and picture analysis to provide a more thorough knowledge of cancer information. For instance, computer vision can extract visual components from medical photographs, whereas NLP algorithms can extract crucial clinical and genetic data from patient records. These data streams can be merged to produce more comprehensive and individual diagnostic reports, enabling healthcare practitioners to make better decisions. By utilising this cutting-edge technology, healthcare providers can enhance the precision, effectiveness, and individualised treatment of cancer diagnosis and therapy. AI-assisted optical imaging has enormous potential to change how cancer care is delivered, eventually enhancing patient outcomes and paving the way for a more promising future in cancer treatment.

## 6. Artificial Intelligence in the Pathology of Cancer

Pathology is a crucial phase in cancer therapy based mostly on optical imaging techniques, such as brightfield microscopy, for detecting bodily abnormalities. Histological sections will be obtained from almost all patients with cancer. The initial stage of AI application in cancer pathology requires rapid and high-resolution capture of histopathological images; otherwise, the evaluation of tumour markers and morphology will be seriously diluted. The traditional visual evaluation of extensive glass slides of tissue slices or biopsies stained with hematoxylin and eosin, immunohistochemistry, or special stains by pathologists has subjectivity, low reproducibility, and misdiagnosis issues. This problem was resolved with the development of a fully automatic digital scanning microscope and the acceptance of whole-slide scanning systems [110]. The AI-enhanced whole-slide imaging system has nearly transformed cancer pathology from simple slice digitalisation to high-throughput photography with automatic image analysis, such as extremely accurate image classification or segmentation and quantitative biomarker evaluation, similar to conventional methods. To date, whole-slide imaging systems have been necessary tools for hospitals, disease control centres, new drug development, and scientific research institutions [111,112,113,114].

The increased use of AI cancer pathology in routine clinical practice has spurred in recent years owing to the application of ML, particularly DL. Traditional image analysis models require labels from the laborious manual annotation of digital slice pictures to be trained, such as metastasis or cancer definitions. Although ML does not require a particular human orientation to specify all rules or parameters in the model, it can employ statistical methods to optimise a model for a particular purpose. DL is a subclass of ML-based image analysis models that hold an intrinsic portion of feature extraction which auto-optimizes learning representations directly from data. In contrast, earlier generations of these models relied on human feature engineering [115]. After the model has been refined using a significant amount of training data, it can be used to forecast reactions or labels in never-before-seen observations by applying identified learning patterns. If so, slice-level labels, such as the presence or absence of malignancy, may be adequate, given the size of the training dataset to train high-performance DL models. Finally, the architecture and characteristics of deep neural networks make it easier to represent extremely complicated and nonlinear patterns in data, and, in many situations, they perform quite well. Recent research has demonstrated that swarm learning (SL), a novel decentralised AI technology, can accurately predict clinically significant genetic changes from images of colorectal tumour tissues. SL-trained AI models have been successfully applied to a large multicentre dataset of histopathology image data containing more than 5000 patients. Additionally, by utilising SL, researchers can train AI models with tiny datasets, which lowers the hardware requirements and makes it possible to train independent AI algorithms for various image analysis tasks without data transfer [116].

The complexity of genetic changes in cancer that impact cellular signalling and the interactions of cells in their microenvironment might affect the biological course of the disease and how it responds to treatment [117]. Much therapeutically important information may be found in digital histopathology images, and AI can use these images to predict molecular alterations from traditional histopathological slides directly. The genotype-tissue expression consortium established successively large deep datasets to map the genetic regulatory effects in various human tissues [118] and showed that multi-tissue, multi-individual data can be used to identify genes and pathways affected by human disease. These efforts were undertaken to provide a systematic understanding of the cellular and biological consequences of human genetic variation and the heterogeneity of these effects among a diverse set of human tissues. AI is increasingly being used to evaluate the severity of cancer and forecast treatment outcomes. For instance, CNN has been utilised to combine data from genomic indicators and histological images to accurately predict the overall survival of patients with glioma, outperforming the current therapeutic paradigm [119]. Machine learning can be used to detect breast cancer nodal metastases [120] in histological or biopsy slides and aid AI in the diagnosis and grading of prostate cancer [121,122,123]. While supervised learning, which has a significant annotation bottleneck, has been the focus of machine learning-assisted pathological recognition, Yu et al. proposed a semi-supervised learning method that can accurately detect and diagnose colorectal cancer from tissue scans as well as pathologists [124]. By using DL for automated segmentation (delineation of boundaries) of histologic primitives (structures) from whole-slide images, novel protocols for kidney biopsy assessment and segmentation of the kidney cortex with multiple histologic stains as well as nearly real-time intraoperative brain tumour diagnosis can be established [125].

Despite these encouraging outcomes, much must be done before trials can be used in clinical settings [68]. The digitalisation of the entire process, including the interconnection of the embedding machine, dehydrator, dyeing machine, and sheet sealing machine, aims to realise precision pathology that is more accurate, efficient, convenient, and low cost. Standardisation of the data format and normalisation method of data analysis may promote the sharing of datasets from different resources that reduce variation in classification accuracy and accelerate model-training maturity [126,127].

## 7. Artificial Intelligence for the Detection and Monitoring of Cancer

**Figure 12:**
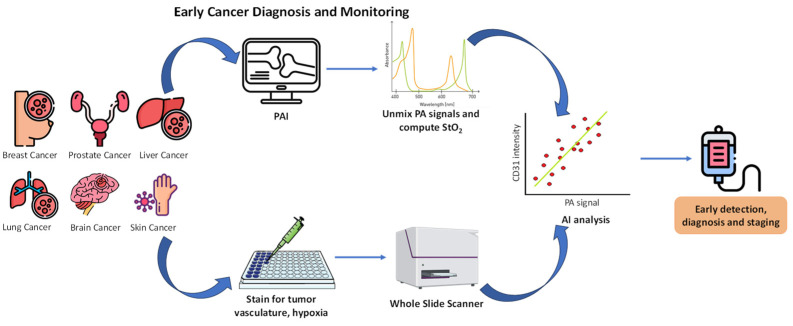
AI automatically extracts richer and more useful information from optical images, whose cancer monitoring and diagnosis show great potential to increase both lifespan and quality of patient life [131].

More than 30 years ago, AI was used to monitor and diagnose cancer despite early attempts that did not produce satisfactory results because of the current situation of constrained computer performance and a lack of data. AI-aided medical imaging for cancer monitoring and diagnosis shows great potential to increase both the quantity and quality of patient life with the remarkable development of AI, particularly DL, with much higher accuracy because it can automatically extract richer and more useful information from the data (Figure 12) [128,129,130].

Radiomics is a procedure that converts digital medical images into data that can be used for analysis. The following analysis is crucial for clinical decision-making. Numerous biomarkers for cancer detection, diagnosis, prognosis, and monitoring are available in radiomics. Additionally, combining the genetic and clinical data can produce a clinical decision report based on evidence. Image acquisition, volume of interest identification, segmentation, feature excision and qualification, database development, classifier modelling, and data sharing are all part of the radiomics process [132].

Integrating AI can boost productivity and reduce errors because of the vast amount of radiomics data. Classical ML algorithms and DL methods are two types of AI methods used in radiomics [133]. ML is a branch of AI that uses statistical techniques to identify hidden patterns. Additionally, DL is used to classify, detect, and segment medical images related to cancer, and its performance is as accurate as that of clinical professionals [134,135,136]. The foundation of conventional ML is specified by the designed feature algorithms with parameters determined by subject matter experts. This technique can improve clinical decision-making and efficiency. However, the flaws are clear. First, the choice of parameters cannot be optimised because they rely on expert knowledge. Second, the established characteristics are not sufficiently flexible to accommodate the various imaging methods. The DL method is a data-driven strategy that can automatically learn from data without needing a human expert. It can detect various types of illnesses automatically. The DL approach, as opposed to the classic ML method, can adjust to different situations and factors and weigh the parameters due to large data to aid clinical decision-making [133].

It has been suggested that radiomics can be used in the framework of AI for cancer monitoring and diagnosis to reveal information that may not be visually obvious to human observers but is nonetheless present in medical images. Beyond what the human eye can see, radiomics uses AI algorithms to extract and analyse numerous quantitative features from medical images. AI-aided radiomics can be successfully combined with multimodal imaging, as well as genomic and clinical data, for clinical diagnosis and monitoring [137]. Aggarwal et al. examined the accuracy of DL for medical imaging in 2021. The meta-analysis results indicated considerable promise but lacked uniform study design and reporting recommendations. Additionally, not many imaging techniques are used in clinics. With the development of optical imaging, a greater variety of monitoring and diagnostic procedures has been developed, including molecular sensitivity, non-ionising, large-scale size, and other characteristics of optical imaging [138]. The clinical decision-making process can be significantly improved by including optical imaging in the radiomics. AI-aided radiomics with optical imaging has shown considerable promise in related cancer diagnosis and monitoring studies. Andre Esteva, who made a significant discovery this year and published it in Nature, created a CNN that is as accurate as or even more accurate than dermatologists in identifying skin cancers. They trained 130,000 medical images covering 2032 diseases using transfer learning techniques with the GoogleNet Inception V3 CNN framework, which had been pre-trained with 1.28 million images. Eventually, this allowed the CNN to classify images as one of the 757 skin diseases, including skin cancer. It also has the benefit of evaluating regular photographs without the need for medical images or pre-processing, as was the case in earlier investigations. Furthermore, the technique is anticipated to be used on mobile devices. It enables people to determine whether a suspicious region is malignant simply by photographing it on their phone rather than visiting a doctor. The authors predicted that 6.3 billion cell phones would be linked to the system globally by 2021, enabling the public to access affordable, precise skin detection services [139].

CNNs are also employed in genetic information analysis to detect gene mutations or changes in gene expression. It is well known that genetic changes in cells lead to cancer. As a result, cancer cells differ from normal cells in their gene sequences and gene expression patterns, which provides a foundation for diagnosis. Genetic testing is the best cancer identification method because genes determine how cells behave. Much work is being done in the scientific community to use genetic information to enhance the accuracy of clinical diagnosis and treatment, such as the continuing “precision medicine” program. The direct detection of genetic abnormalities can result in the early detection of cancer. However, medical imaging is cheaper than mutation testing or gene expression data. Additionally, the message from genetic testing is much more nuanced, and more research is needed to determine the precise connection between genes and cancer than is now available. As a result, there is currently little pertinent research in this field. Notably, Yuan created DeepGene in 2017, a deep neural network-based technology that analyses patient genetic mutation data to determine the type of cancer the patient belongs to. DeepGene employs a deep neural network to identify cancer types after removing unnecessary genes and reducing data sparsity from gene sequencing data. Despite having greater accuracy than other earlier techniques, such as SVMs and Naive Bayes, it is still only approximately 60% accurate [140]. To identify stomach, lung, and breast cancers based on gene differential expression data, Xiao developed a method incorporating numerous machine learning models and deep neural networks. However, the accuracy was not high [141].

A significant obstacle to treatment that only targets primary tumours is that the primary location of tumour formation in approximately 1.2 percent of cancers cannot be identified, and the median overall survival was just 2.7 to 16 months. To tackle this challenging issue, Mahmood et al. created TOAD. This DL-based tumour-origin prediction system can simultaneously determine whether a tumour is primary or metastatic and predict where it originated. To construct an AI model for an unknown primary cancer, the model was trained using whole slices of gigapixel tumour caseology from more than 22,000 cancer cases. TOAD was found in roughly 6500 known primary cases and examined for increasingly complex metastatic cancer cases. For tumours with a known primary origin, the model correctly predicted the diagnosis in the top three positions 96% of the time and identified cancer 83% of the time. The TOAD test was found to agree with the pathologist’s report in 61% of cases and with the top three predictions in 82% of cases in 317 cases of primary unidentified cancer with differential diagnoses [142], suggesting that TOAD can aid in the diagnosis of patients with complex metastatic cancer.

AI is also used to evaluate various medical photos to identify malignancies, such as osteosarcoma, head and neck, bladder, brain, and oral cancer, in addition to the previously-mentioned cancer categories.

## 8. Artificial Intelligence for the Diagnosis and Treatment of Cancer

**Figure 13:**
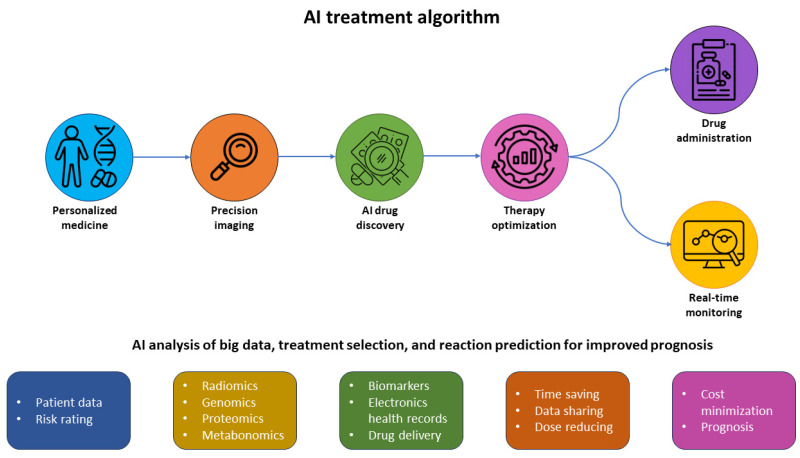
The application of AI in cancer treatment and prognosis [131].

AI enables quick and affordable access to new medications and treatments. AI-integrated diagnostics and biological big data with genomics, proteomics, metabolomics, and radiomics provide powerful support for clinical decision-making and cancer treatment planning, monitoring, administration, and optimisation based on outcome prediction [143]. Over the following several years, AI appears to be promising for use across boards in the medical sector (Figure 13).

Based on patient data, AI can improve tailored cancer treatment. It is vital to design individualised treatments based on patient data because the efficacy of the same medication may differ from patient to patient. AI tools, ranging from machine learning to neural networks, can accelerate the discovery of new drugs, precisely match patients with the right clinical medicines using biomarkers, and truly customise cancer treatment utilising patient data. RL is highly effective in drug design. Instead of the customary timeframe of approximately a year, reinforcement learning successfully designed a novel compound in just 21 days by utilising rewards and penalties to train the algorithm to obtain the required drug structure. Additionally, the subsequent pharmacokinetic features of the proposed compounds showed that they are capable of causing drug exposure (drug exposure is the term used to describe the degree of exposure to medicines, including time and intensity, as measured by AUC, Cmax, Tmax, etc.). The next round of lead compound evaluations can apply an effectiveness threshold. AI can also be used to recruit patients in a hierarchical manner as well. Patient outcomes improved when biomarkers were used in trial recruitment instead of more conventional stratified data, such as pathology or treatment response. Electronic health records (EHRs), patient biomarker data, and patient stratification may further affect trial outcomes. AI is crucial for the delivery of cancer medicines. Drug-sensitive tumour cells were eliminated at the highest tolerable dose. However, resistant cells may ultimately lead to therapeutic failure. Game theory is being investigated as a solution to this problem to prevent drug-sensitive cells from outnumbering drug-resistant cells, which have a high energy cost. This is referred to as adaptive therapy, which may extend the duration of the therapeutic effect by preserving a threshold of drug-sensitive tumour cells to thwart the spread of drug-resistant cells.

There are disadvantages, but AI opens the door to the future of cancer treatment. A considerable improvement in patient outcomes is unlikely, for instance, if AI-optimized drugs are combined with other therapies unfavourably or if the medication is prescribed incorrectly. It will take full integration of AI across domains, including research, development, and management, to overcome this hurdle. Increasing the resolution of tailored care by designing customised programs incorporating several treatment modalities is an example of a potential downstream application. For instance, AI may be combined with AI-driven medicine delivery to optimise the radiation dose and maintain excellent tumour size control. Ultimately, full integration of AI into clinical oncology practice may enhance drug accessibility and lower medical expenses. It is becoming increasingly clear that AI can alter clinical standards for cancer care as it continues to be validated and avenues for general practice are found [144]. We sought the thoughts of four experts on how we may start implementing AI while ensuring that standards are upheld to revolutionise cancer diagnosis, prognosis, and treatment of cancer patients and to promote biological discovery [145].

Precise diagnosis and sensible decision-making are essential for cancer therapy and prognosis, which necessitate constant monitoring of dynamic tumour microenvironment changes in a highly precise manner while considering many factors. Artificial intelligence-assisted optical imaging, which noninvasively captures tumour phenotypes and suggests potential pathophysiological changes based on biomarkers, is crucial for stratifying tumour malignancies, predicting therapeutic outcomes, and analysing tumour heterogeneity in both qualitative and quantitative ways, which is closely related to prognosis. While traditional tumour surveillance is frequently restricted to tumour volume, AI might track the entire course of tumour treatment over time and record a staggering array of tumour variables that represent tumour progression and efficacy in real-time [146]. Machine learning can be used to mine imaging data with molecular and pathological data to improve the ability of doctors to identify, treat, and manage cancer. This information could be used for screening, developing targeted drugs, optimising personalised therapy regimens, and predicting cancer outcomes [147]. Tumour-related factors that generate significant changes in the emergence and development of tumours are frequently characterised using molecular data such as genome, DNA methylation, mutation, m6A, and single-cell sequencing data. An individual’s likelihood of having a specific cancer can be determined by identifying risk factors, and doctors can then advise patients to follow preventive treatment methods based on this information. A novel cancer AI survival analysis system was created by Liang et al. to provide cervical carcinoma patients with individual mortality risk predictive curves based on three different AI algorithms, which could provide mortality percentages at particular time points and explore the actual treatment benefits under various treatments in four stages, helping patients choose the most personalised treatment [148].

The future of treatment planning includes AI-based automated radiation planning systems, which have been implemented in several cancer sites. In addition, volumetric modulated arc therapy (VMAT) has gradually become the standard radiotherapy method because postmastectomy radiation therapy increases overall survival while lowering the likelihood of local recurrence. To guarantee plan quality and boost clinical effectiveness, Jiang et al. developed an AI-based automated treatment-planning technique for postmastectomy VMAT [149]. The emergence of AI has also provided fresh life for liver cancer surgery, more tailored therapy options and patient recovery chances [150]. Along with immunotherapy, additional treatments (such as targeted therapy and neoadjuvant chemotherapy) have shown notable clinical success in particular populations, necessitating the development of precise predictive assays to guide patient selection. Big data and AI can fulfil this requirement [151]. AI is also used in nanomedicine design by optimising material properties following projected interactions with the target medication, biological fluids, immune system, vasculature, and cell membranes, all affecting therapeutic efficacy [152]. A possible candidate for cancer management and prevention applications is a triboelectric nanogenerator with AI-based systems [153].

The research team assessed histopathological patterns in 17,355 histopathology pictures from 28 different cancer types using deep transfer learning and connected these patterns with corresponding genomic, transcriptomic, and survival data. Genome-wide duplications across cancer types, single chromosome aneuploidy, lesion amplification and deletion, and ubiquitous features in driver gene mutations have all been associated with a significant number of recurrent genetic aberrations in a variety of cancer types. AI can enhance cancer diagnosis, prognosis, and treatment, as demonstrated by the significant potential of computer vision to identify the molecular foundation of tumour histology [154].

AI is advantageous for solving the most challenging issue of extremely high clinical failure rates through prediction of the toxicity of candidate drugs to animals and humans. One example of use is the pharmacokinetic/pharmacodynamic index, which can save billions of dollars, improve R&D efficiency, and shorten several years of drug discovery and verification, developed by many top pharmaceutical companies, such as GlaxoSmithKline, which have gradually made this technology a reality. On the other hand, negative data and unpublished experimental records were mined through machine learning to revalidate and optimise drug synthesis routes based on decision trees and support vector machines, which outperformed conventional human strategies and accurately predicted conditions for new organically templated inorganic product formation with an 89% success rate [155]. The precise prediction of protein structure by AlphaFold2 is another illustration of how AI may revolutionise industries and alter the planet. Because biological networks can efficiently preserve and quantify the relationships between cellular system components that underlie human diseases such as cancer, AI is a cutting-edge method for identifying novel anticancer targets and discovering innovative therapeutics. We may examine the connection between network characteristics and cancer using artificial intelligence models, which provide a quantitative foundation for finding possible anticancer targets and new medication candidates [156]. Each year, hundreds of new cancer therapeutics enter clinical trials; however, less than 4% of these treatments are ultimately authorised by the U.S. Food and Drug Administration (FDA). The fundamental issue is that we do not completely understand how or why particular malignancies respond to treatment, even though other factors may have contributed to this outcome. As a result, it is currently impossible to match optimal medicine combinations to appropriate patients. However, the emergence of AI may provide humans with advantages. Most machine learning models are “black boxes”, which may be accurately optimised without having to know or care about the biology underlying the predictions. DrugCell, a new artificial intelligence (AI) system that enables tumours to be matched with ideal treatment combinations, has recently been developed. After entering information about the tumour, DrugCell returns the most effective drug, the biological mechanism that governs the reaction of the drug, and the optimal drug combination. DrugCell uses the hierarchy of human cell biology and the inner workings of models to predict the response to any treatment for any malignancy and to create potent combination therapies. The results demonstrated that DrugCell could predict the cell line response to therapy with high accuracy (Spearman correlation coefficient for all cell line-drug pairs: 0.80). Additionally, the anticipated combination increases progression-free survival in xenograft tumour models made from actual patients and can stratify clinical outcomes in patients with ER-positive breast cancer. More than 500,000 cell line/drug combinations have been used to train DrugCell on how more than 1200 tumour cell lines react to approximately 700 FDA-approved medications and investigational therapeutic agents. Although 1200 cell lines are a wonderful place to start, they do not fully capture the diversity of cancers. The research group is currently exploring various medication structures and adding single-cell data. They also intend to collaborate with previously conducted clinical studies to integrate DrugCell into diagnostic tools and evaluate it prospectively in practice [157]. AI has shown potential application value in a novel computational framework called EagleC, which uses chromatin capture technology to identify genomic structural variations based on DL. This framework uses DL to identify genomic structural variations [158]. Examples of the use of theranostic agents with AI are shown in Table 2.

## 9. Clinical Trials for Theranostic Agents

Although the development and use of nanoparticles as possible theranostic agents are still relatively new, the U.S. Food and Drug Administration (FDA) has granted IND approval for a small number of these to be tested in clinical trials. For instance, it has been suggested that combining radioisotopes and quantum dots can improve the sensitivity and accuracy of detecting cancerous cells while also enabling targeted therapies [162,163]. Prior research by Benezra et al. (2011) showed that Cornell dots (C dots) conjugated to the cyclic peptide (Arg-Gly-Asp-Tyr) were capable of reliably identifying the presence of cancer cells that expressed the anb3-integrin in a human melanoma xenograft model [162]. Malignant cells with active angiogenesis overexpress integrin anb3 [162,163]. Due to the selective nature of the aforementioned imaging probes, radioisotope-labelled particles were created, which could aid in precise diagnosis and focused therapy. Positron emission tomography (PET) imaging using carbon dots labelled with ^124^I for the cyclic peptide (Arg-Gly-Asp-Tyr) revealed the exact diagnosis and tailored therapy for melanoma patients with minimal side effects [163]. Furthermore, phase III randomised NAPOLI-1 (NCT01494506) research found that using theranostic nanoparticles, such as liposomes, increased the survival of cancer patients receiving treatment [164,165]. Patients with metastatic pancreatic adenocarcinoma who received liposomal irinotecan together with leucovorin and 5-fluorouracil (nal-IRI5-FU/LV) showed a considerably extended survival rate, with few side effects and toxicity [164,165]. Table 3 provides an overview of the numerous ways in which theranostic drugs have been used in clinical trials.

## 10. Summary

This article discusses the application of photodynamic therapy (PDT) in cancer diagnosis and treatment, focusing on the design and use of photosensitizer-based theranostic agents. The combination of diagnostics and therapy in a single theranostic agent allows the precise targeting and destruction of cancer cells with minimal impact on healthy tissues. Theranostic agents are designed to be active in a specific tumour microenvironment, which increases their therapeutic efficacy. Photosensitisers, such as BODIPY, Ir(III) complexes, and quantum dots, show high photochemical stability and specific targeting of cancer cells. These compounds enable cancer imaging and provide effective treatment by generating ROS or heat when exposed to light. Studies have shown that small structural changes in theranostics can significantly affect selectivity and therapeutic efficacy. Examples include differences in the oligoethylene glycol chain length of BODIPY compounds, which affects their intracellular localisation and photocytotoxic effects. Integrating AI techniques such as natural language processing (NLP) and image analysis with traditional diagnostic methods can significantly improve cancer diagnosis and treatment accuracy and efficiency. AI allows for more complex and individualised analysis of patient data, leading to better treatment planning.

The development and application of photosensitizer-based theranostic agents offer promising opportunities for precise cancer treatment and diagnosis by combining imaging and therapy in a single approach. The integration of AI technologies further enhances the potential of these approaches, leading to more individualised and effective cancer treatment strategies.

## Figures and Tables

**Table 1:** Quantum dot-based cancer theranostics [66].

Quantum Dots Agent	Applications		References
Pegylated Black Phosphorus Quantum Dots (PEGylated BPQDs)	Suppressed the growth of tumours in 4T1-tumour-bearing mice without the presence of adverse effects	In vivo	[69]
Quantum dot-based micelle conjugated with EGFR antibody and loaded with aminoflavone	Induced regression of MDA-MB-468 triple-negative breast cancer cells by targeting EGFR	In vitro; In vivo	[59]
Lipid micelles co-loaded with paclitaxel/quantum dots and EGFR antibodies/EGFR aptamers	Induced cell cycle arrest at the G2/M phase and triggered apoptosis in treated S174T human colorectal cancer cells by targeting EGFR	In vitro; In vivo	[4]
Iron oxide-bismuth oxide-graphene quantum dots (GQDsFe/Bi)	Induced apoptosis in treated MCF-7 breast cancer cells upon laser irradiation	In vitro	[58]
Graphene quantum dots-Candida parapsilosis biosurfactant conjugates	Decreased the viability of MCF-7 breast cancer cells in a dose- and time-dependent manner	In vitro	[60]
Graphene quantum dots/photosensitiser/CpG oligonucleotides hybrid attached to dual magnetic resonance/fluorescence imaging probes (PC@GCpD [Gd])	Hindered the growth of EMT6 murine mammary cancer cells via the upregulation of relevant immune response for the destruction of cancer cells	In vitro; In vivo	[70]

**Table 2:** Examples of therapeutic compounds used in PDT and PDD, in the analysis of which AI has been involved.

Name	Application	References
Photofrin	**Image Analysis:** AI analyses images from computed tomography (CT) or magnetic resonance imaging (MRI) to precisely determine the location of tumours and Photofrin accumulation. This enables more accurate therapy planning and optimisation of lighting parameters. **Progress Monitoring:** AI algorithms track changes in tumour size and response to treatment based on sequential images, allowing dynamic adjustment of the therapy plan.	[159]
Aluminium Phthalocyanine (AlPc)	**Light Dose Optimization:** AI helps simulate and optimise light delivery parameters such as wavelength, intensity, and duration based on individual patient characteristics and photosensitiser distribution. **Predictive Modelling:** AI-based predictive models can forecast patient response to treatment using AlPc, allowing personalised therapy.	[160]
Temoporfin (mTHPC)	**Therapy Planning:** AI uses genetic, metabolic, and imaging data to predict how temoporfin will distribute and accumulate in cancer tissues. This allows for more precise therapy planning and execution. **Dosimetry Automation:** AI automatically calculates light dosimetry, ensuring the correct amount of energy is delivered to activate the photosensitiser without excessive damage to surrounding tissues	[161]

**Table 3:** Clinical trials of theranostics in cancer.

Theranostic Agents Used in Clinical Trials	Outcomes	Clinical Trial	References
“C dots” (Cornell dots) labelled with ^124^I (PET imaging) and conjugated with cRGDY peptides (targeting agent) (^124^IcRGDYePEGeC dots)	Since cRGDY targets integrin anb3, which is overexpressed on endothelial cells involved in angiogenesis, vascular remodelling and solid tumour cells, the accumulation of ^124^I-cRGDYePEGeC dots on cancer cells could be observed using PET. This method can be used for the selection of patients who require integrin-targeted treatments, imaging and diagnosis of tumour cells and neovasculature and to monitor the progress and efficiency of a particular treatment	NCT01266096	[163]
Paclitaxel-loaded polymeric micelle and cisplatin	Paclitaxel-loaded polymeric micelle at a dose of 230 mg/m^2^ in combination with cisplatin was well tolerated with minimal toxicity in non-small cell lung cancer patients when given in a 3-week cycle	Phase II trial	[166]
NK012 polymeric micelle	The anticancer activity of NK012 was tested against unresectable, metastatic, and recurrent colorectal cancer patients. Treated patients with a history of oxaliplatin-based therapy demonstrated side effects such as diarrhoea and neutropenia.	Phase II trial	[167]
^177^Lu-PSMA-617 and ^68^Ga-PSMA-11	Accumulation and prolonged retention of ^177^Lu-PSMA-617 in tumour tissues compared to normal cells in treated metastatic prostate cancer patients resulted in decreased prostate-specific antigen (PSA) levels, which are overexpressed in prostate cancer patients. The accumulation of ^177^Lu-PSMA-617 in tumour cells was observed using 68Ga-PSMA-11 via PET.	ANZCTR12615000912583	[168]
^68^Ga-PSMA-11	Accurate and precise diagnosis of recurrent prostate cancer in prostate cancer patients using ^68^Ga-PSMA-11 using positron emission tomography (PET) were achieved, suggesting the importance of this theranostic agent to aid in the diagnosis of prostate cancer patients.	(NCT02940262; NCT03353740)	[169]
^177^Lu-PSMA-617	This study is still ongoing. However, it has been hypothesised that metastatic castration-resistant prostate cancer (mCRPC) patients treated with ^177^Lu-PSMA-617 would have a longer lifespan and less cytotoxicity towards the surrounding normal cells compared to cabazitaxel chemotherapy.	Phase II trial (NCT03392428)	[163]
STARD3	This study aims to verify the overexpression of STARD3 in both early and advanced CRC patients’ derived tissues to identify the pathways underpinning tumourigenesis and cancer progression in which STARD3 is involved. Moreover, its role as a dynamic biomarker of treatment response and its role in treatment sensitivity will be explored.	NCT06136949	[170]
[^68^Ga]Ga DOTA-5G and [^177^Lu]Lu DOTA-ABM-5G	This is a Phase I study to evaluate the safety and efficacy of the [^68^Ga]Ga DOTA-5G and [^177^Lu]Lu DOTA-ABM-5G theranostics pair in patients with metastatic cancer. [^68^Ga]Ga DOTA-5G PET/CT will be used to identify and stratify patients eligible for (and most likely to respond to) the [^177^Lu]Lu DOTA-ABM-5G therapy.	Phase I trial NCT06389123	[171]
18F-PSMA PET/CT	The aim is to evaluate whether PSMA-directed in vivo imaging can also be applied to GEP-NEN patients to determine if (i) biopsy-derived tissue of newly diagnosed patients exhibits a PSMA expression profile, (ii) PSMA-PET shows upregulated PSMA expression in vivo, (iii) such a molecular imaging approach identifies more disease sites relative to conventional imaging, and (iv) if the PSMA PET signal predicts further clinical course and outcome under guideline-compatible treatment.	NCT05547919	[172]
